# Is violence associated with increased risk behavior among MSM? Evidence from a population-based survey conducted across nine cities in Central America

**DOI:** 10.3402/gha.v7.24814

**Published:** 2014-10-23

**Authors:** Jennifer Wheeler, Katherine Anfinson, Dennis Valvert, Susana Lungo

**Affiliations:** 1 Population Services International, Washington, DC, USA; 2 Association PASMO, Guatemala City, Central America

**Keywords:** HIV, key populations, MARPS, MSM, transgender women, HIV risk, violence, IPV, Central America

## Abstract

**Background/Objective:**

There is a dearth of research examining the linkages between violence and HIV risk behavior among men who have sex with men (MSM), including those who identify as transgender women (TW), particularly in Central America where violence is widespread. In this paper, we use population-based survey results to independently examine the correlations between physical, emotional and sexual violence and HIV risk behavior among MSM populations in five countries in Central America.

**Design:**

As part of USAID's Combination Prevention for HIV program in Central America, PASMO conducted population based surveys using respondent-driven sampling (RDS) in nine cities in Guatemala, El Salvador, Nicaragua, Costa Rica, and Panama. Initial seeds were recruited using the following criteria: individuals who represented subgroups of MSM by self-identification (homosexual vs. heterosexual or bisexual vs. transgender), social economic strata, and by sex work practices. This study examines the association between violence and 1) HIV risk behaviors relevant to the study populations; 2) protective behaviors; and 3) reported STIs. Individualized RDS estimator weights for each outcome variable were calculated using RDSAT software, and logistic regression analysis was used to determine associations between different forms of violence and the outcome variables.

**Results:**

MSM who experienced physical violence were more likely to be engaged in transactional sex (OR: 1.76 [1.42–2.18]), have multiple partners in the past 30 days (OR: 1.37 [1.09–1.71]), and have engaged in sex under the influence of alcohol or drugs (OR: 1.51 [1.24–1.83]). Both physical violence and psychological/verbal violence were also associated with reporting STI symptoms or diagnosis within the past 12 months (OR: 1.72 [1.34–2.21] and 1.80 [1.45–2.23]). The effects of violence on the outcomes were observed after controlling for other risk factors. Transgender women were 3.9 times more likely to report engaging in transactional sex. Respondents who were heterosexual, bisexual, or transgender were also more likely to both report multiple partnerships (OR: 1.44 [1.07–1.96], 1.99 [1.67–2.38], 1.79 [1.37–2.33], respectively) and more likely to report engaging in sex under the influence of alcohol or drugs (OR: 1.52 [1.15–2.01], 1.38 [1.17–1.63], 1.47 [1.16–1.87], respectively), as compared to those identifying as homosexual.

**Conclusion:**

Violence experienced by MSM and TW is widespread in Central America. The experience of violence is shown in this study to be independently associated with risk behaviors for HIV infections. Further research and studies are needed to identify the effects violence has on HIV risk behavior among this under-researched population to improve targeted HIV prevention interventions.

Nearly 150,000 people are living with HIV/AIDS in Central America. While HIV prevalence among the general population is below 2% (1), higher prevalence rates are found among key vulnerable populations defined as men who have sex with men (MSM), female sex workers (FSW), and MSM who identify as transgender women (TW) (2, 3). The greatest HIV burden is found among TW – country-level estimates range from 7.5% in Belize to 28.6% in Honduras (1), compared to 19.1% globally (4). In this paper, we refer to MSM as men who engage in sexual activity with other men regardless of how they identify themselves (with regards to sex or gender) (3). Several studies concur that violence may increase an individual's risk for HIV infection through forced or coercive sexual intercourse, irrespective of relationship type (same sex or male-female) (3, 5–7). Studies reviewed have also found a relationship between various forms of violence, including sexual violence, intimate partner violence, physical violence, community violence, and risky sexual behavior or STIs (8, 9). In both middle-and high-income countries, exposure to intimate partner violence, including controlling behavior of a partner, physical violence, and sexual violence, is associated with high-risk sexual behavior, including multiple and concurrent sexual partnerships, substance abuse, and transactional sex, thereby heightening the risk for HIV and other STIs (10–12). Globally, intimate partner violence is often seen as a direct consequence of gender power inequities, both at the community and interpersonal levels that increases the vulnerability and decreases decision-making power among victims (13).

Violence is widespread throughout Central America, with El Salvador, Guatemala, and Honduras facing some of the worst statistics in Latin America for homicide, robbery, and victimization rates. Close to 16% of adults in Guatemala and El Salvador report being victims of violent crime within the previous 12 months (14). Domestic Violence is also high, with estimates of physical abuse ranging from 16 to 39% across five countries in Latin America (15). The most recent data on intimate partner violence in Central America comes from Guatemala, where 24.5 and 12.3% of women report ever being physically or sexually abused and 16% of men report perpetrating physical violence within a marital relationship (16). Within a socio-ecological framework, violence within the community precipitates other forms of violence (17). Previous studies have shown that societal level violence and indirect violence (witnessing violence against others) are significantly associated with the experience and perpetration of intimate partner violence (18, 19). Indirect violence is also hypothesized to have independent effects on vulnerability to and risk of HIV (18), and the experience or perceptions of neighborhood violence are found to have significant effects on domestic violence (19).

Closely related to this context of endemic violence is the ‘machismo’ culture and widespread homophobia found in Central America. Results from a regional study found that close to 40% of respondents believe that ‘God’ punishes homosexuals and sex workers with AIDS for their way of life, and 38.5% of respondents agree that people have the right to assault transgender and transvestite individuals (20). Individuals associated with homosexual behavior in Central America are often hidden and exposed to social marginalization, stigma, targeted homophobia, and discrimination (1, 21). In social and cultural contexts where homophobia or transphobia is prevalent, MSM and TW are at an even greater vulnerability due to structural factors (2, 22). Structural factors, including social, economic, organizational, and political inequities (23) lessen MSM and transgender women's ability and motivation to report violence, negotiate safer sexual practices (24), seek healthcare, and/or disclose sexual orientation to health care providers (25).

The experience of violence is found to be more common in MSM than among heterosexual men, and possibly women (26). Data collected from a study in Thailand and Mexico among MSM and TW showed that at least 50% of MSM and over 65% of TW screened through HIV clinical services and drop-in centers experienced emotional, physical, or sexual violence. TW, in particular, were found to experience greater levels of violence in both countries (22). However, there is a dearth of research examining the linkages between violence and HIV risk behavior among MSM and TW due, in part, to the highly stigmatized nature of homosexual behavior (3, 7, 27, 28).

The associations between sexual identity, violence, risk of-and vulnerability to-HIV infection are complex. Available literature shows that both heterosexual and lesbian, gay, bisexual, and transgender (LGBT) populations who experience intimate partner violence are at greater risk for HIV/STIs (5). Moreover, due to their position of vulnerability and disempowerment, MSM and TW who experience violence are less likely to negotiate safer sex practices, often engage in riskier behavior that further increases their risk of HIV/STIs, and face greater obstacles in accessing health services (22, 27). The magnitude of violence experienced by MSM and its association with HIV are inconsistently addressed and poorly understood by healthcare, legal, and social services (29). A US-based study conducted in Los Angeles among professionals affiliated with health, psychosocial, and legal services for survivors of violence report that their respective agencies are ill-equipped to address the needs of LGBT populations (30). Previous experience with stigma and discrimination from law enforcement is a barrier for MSM in the pursuit of legal recourse in incidents of violence (31).

The purpose of this paper is to contribute to the growing evidence that looks at the association of violence with HIV/STI risk behavior among MSM populations in Central America. In this paper, we use population-based survey results to independently examine not only the correlation of physical violence but also verbal/psychological and sexual violence on HIV risk behavior among MSM populations across nine cities in Central America. Our data are inclusive of TW as the broader group of MSM and we are able to examine the relationship between self-identification and risk behaviors under study. Through this study, we are able to quantify experiences of violence among this group, which is currently understudied (3) and very relevant in terms of public health and human rights.

The theoretical framework used in this study hypothesizes that MSM populations in Central America who report experiences of violence are more likely to engage in risky sexual behavior further increasing their risk of acquiring HIV and STIs as evidenced in our review of literature (13, 26, 28). This framework extends an individual-level behavioral model to include situational factors, such as the experience of violence and forms of social vulnerability, to explain engagement in HIV risk or protective behaviors (13).

Of particular interest to our analysis is the experience of different forms of violence, either physical or psychological/verbal, and sexual self-identification. Measures of sexual identity and ‘outness’ were previously proposed as determinants of both violence and HIV risk behaviors in MSM (7, 12, 32). Studies have shown differential levels of HIV testing among categories of self-identification (33) and associations between identity and HIV risk behaviors that are both direct and indirect (operating through substance abuse, battering, aversive emotions, and HIV alienation) (32). We examine the experience of different types of violence and their associations with reporting multiple partners, engagement in transactional sex, use of alcohol and drugs during sexual relationships, and reported STI symptoms. We argue that further research and studies are needed to deepen our understanding of the correlation between violence experienced by MSM and TW populations as a mechanism that leads to increased HIV risk behaviors and to improve HIV prevention interventions.

## Methods

### Purpose of the study

In 2011, Population Services International's regional affiliate – the Pan American Social Marketing Organization (PASMO) – conducted population-based surveys, with funding support from USAID and PEPFAR's Combination Prevention for HIV program in Central America. To evaluate and improve HIV prevention efforts among MSM and TW. These population-based surveys were designed by the PASMO/PSI Regional Research and Metrics team and implemented by CID Gallup. This study provides estimates of different forms of violence among understudied groups, previously unmeasured.

The surveys were conducted using respondent-driven sampling (RDS) in nine cities in Guatemala, El Salvador, Nicaragua, Cost Rica, and Panama. The sites included in this study were selected because they were key target locations of the Combination Prevention for HIV program. The total sample size for all cities combined was 3,949; sample sizes per city are shown in Table 1.

**Table 1:** RDS recruitment and sample sizes by city

Country	Cities	Number of seeds	Waves (max)	Total sample
Guatemala	Quetzaltenango	5	9	308
	Guatemala City	3	12	487
El Salvador	San Salvador	2	15	436
	Santa Ana	2	13	298
Nicaragua	Chinandega	5	9	293
	Managua	4	12	489
Costa Rica	San Jose	8	13	752
Panama	Panama City	6	19	530
	Colon	7	7	356
	Total			3,949

### Study participants recruitment

RDS is widely used to sample hidden or hard-to-reach populations such as MSM and TW. This sampling methodology allows researchers to measure HIV risk behaviors and HIV/STI prevalence and helps to attain a representative sample when studying a population without a sampling frame (34). The use of RDS is preferred over snowball sampling as it reduces biases through a more controlled peer and social network recruitment while using statistical weights to provide unbiased population estimates (35, 36).

In this study, RDS was used to recruit a sample of individuals in each of the selected cities. The initial group of individuals, known as ‘seeds’, was selected using the following criteria: individuals who represented subgroups of MSM by self-identification (homosexual vs. heterosexual or bisexual vs. transgender), socio-economic strata (low-, middle- and high-income), and by sex work practices (charged for sex in the past year or not). These initial groups began the recruitment process using a ‘chain referral’ methodology (35). RDS uses a dual incentive system to drive the chain referral methodology by having seeds recruit peers from within their social network (36, 37). In the PASMO studies, participants received a US$5.00 incentive upon being interviewed and incentives of equal value for recruiting a maximum of three eligible peers into the study.

### Outcomes of field work

The total number of seeds used in each city ranged from two to eight. The process began by selecting two seeds per country, and then using additional seeds when recruitment chains terminated. As indicated in Table 1, in El Salvador and Guatemala City, the samples were achieved using two or three seeds and the highest number of waves ranged between 12–15. Other countries required additional seeds, but these were able to achieve recruitment waves up to 7–19. RDS is based on the assumption that with a sufficient number of recruitment waves, the composition of the sample becomes independent of the initial seeds (35) and is said to reach ‘equilibrium’. In each site, we used RDSAT v 6.0 to calculate measures of homophily, the maximum number of waves needed to reach equilibrium, and the equilibrium proportions for three key outcomes (condom plus water-based lubricant use, multiple partnerships, and sex under the influence of alcohol/drugs). In all sites, the maximum number of waves and the average number of waves surpassed the estimated number of waves required to achieve equilibrium. Diagnostic measures suggested that the samples did not demonstrate a high level of homophily (the principle that people associate and recruit people who are similar to themselves (38).

### Ethical considerations

Ethical approval was granted by PSI's Research Ethics Board (REB) in Washington, DC. The study was also submitted to and approved by the Ministry of Public Health in Belize, El Salvador, Guatemala, and Nicaragua, and by the local ethics committee in Panama and Costa Rica; HIV and AIDS National Program in Panama; and the University of Medical Science in Costa Rica.

To ensure protection of subjects participating in the study, no printed information was given unless requested. Also, references to sex or MSM activities were not included in the RDS vouchers. Verbal informed consent was obtained for each participant by interviewers prior to conducting the interview, with the exception of Costa Rica, where the local IRB required written informed consent. The interview took place at the study site or at a location indicated by the study participant.

### Measure of interest

Four measures of violence were collected as part of this study. These were defined as:

- Psychological violence: having felt threatened, in fear or put in danger by somebody in the past 12 months;
- Verbal violence: having been yelled at, insulted, humiliated, or made felt inadequate by somebody in the past 12 months;
- Physical violence: having been slapped, punched, hit, or harmed physically by somebody in the past 12 months; and
- Sexual violence: having been forced or coerced to have sexual relations against their will in the past 12 months.

This study examines the association between violence and: 1) HIV risk behaviors relevant to the study populations; 2) protective behaviors; and 3) reported STIs. These outcomes are defined as:

- Transactional sex: reporting partners with whom the respondent received payment for sex in the past 30 days.
- Sex under the influence of drugs or alcohol: reporting sex under the influence of alcohol or drugs with any type of partner in the past 30 days.
- Multiple partners: reporting more than one sex partner in the past 30 days.
- Condom plus lubricant use: reporting joint use of a condom and lubricant at the last sexual encounter.
- Reported STI diagnosis or symptoms: reporting abnormal or excessive genital discharge, reporting an ulcer, sore, pustule, or excessive genital itching; and/or having an STI diagnosis in the past 12 months.

## Analysis

Weighted univariate point estimates are presented for each city, and unweighted estimates are presented for all cities combined (under the regional column in Table 2). Individualized RDS estimator weights for each outcome variable were calculated using RDSAT v 6.0 software and exported to STATA version 12.0. Individualized weights are calculated using the respondent's network size and composition using the dual component estimate. The RDS methodology and accompanying software was designed for point estimation but does not lend itself to multivariate analysis (39, 40). The unweighted multivariate findings thus cannot be generalized beyond the population under study; however, they still provide important insights regarding the relationships between violence and HIV risk behaviors that can inform program interventions and future research.

**Table 2:** Weighted univariate distributions of violence, risk behaviors, condom and lubricant use, and reported STIs among MSM in Central American Cities

	Guatemala		El Salvador		Nicaragua		Costa Rica	Panama		Regional (unweighted)
				
	Guatemala	Quetzaltenango	San Salvador	Santa Ana	Managua	Chinandega	San Jose	Panama	Colon
N	464	300	428	294	471	289	733	518	317	3,814
Experienced psychological violence in the past 12 months	35.57	20.93	27.53	23.39	28.90	10.06	20.00	10.74	12.17	22.26
Experienced verbal violence in the past 12 months	44.87	28.13	32.48	17.65	39.67	10.20	28.68	7.64	6.12	26.19
Experienced physical violence in the past 12 months	25.04	12.60	16.00	8.39	18.30	3.93	14.82	4.57	6.39	13.43
Experienced sexual violence in the past 12 months	5.38	6.87	6.50	4.06	7.35	1.96	2.11	4.03	4.20	5.11
Transactional sex in the past 30 days	40.33	12.89	31.83	18.03	19.77	23.26	35.81	17.71	17.30	27.03
More than one partner in the past 30 days	76.27	59.95	71.85	76.17	54.87	80.00	70.94	56.22	77.79	69.46
Sex under influence of drugs or alcohol	44.50	39.11	36.85	36.39	25.37	34.69	45.54	27.77	56.99	40.59
Condom and lubricant use at last sex	60.35	58.07	35.15	37.75	39.03	38.27	44.94	62.96	49.05	48.08
Had an STI or symptoms in the past 12 months	17.48	15.24	11.33	13.44	6.01	5.22	7.54	12.94	34.55	14.03

*Calculated for an analytical sample with no missing values on all covariates.

Logistic regression analysis was used to determine whether there are associations between the five outcome variables and violence, after adjusting for other covariates. Covariates were determined on a review of the literature and availability of data measured as part of the study, and include: 1) socio-demographic variables, such as age, marital status, having one or more children, socio-economic status (SES), educational attainment, ethnicity, and city of residence and 2) other variables measuring social vulnerability, including self-identification (whether the respondent self-identifies as heterosexual, homosexual, bisexual, or transgender) and housing insecurity (the number of times within the past 12 months that the respondent has changed residences) (7, 12, 32, 33, 41, 42). Table 3 presents the unweighted distribution of these covariates by city. Measures of verbal and psychological violence were combined to form a single variable because of their high correlation and conceptual overlap. Measures of sexual and physical violence were combined, as the distribution of sexual violence was too low to be included in multivariate model. Associations are presented as odds ratios and with their respective confidence intervals.

**Table 3:** Socio-demographic characteristics among MSM in Central American cities

	Guatemala		El Salvador		Nicaragua		Costa Rica	Panama		Regional
				
	Guatemala	Quetzaltenango	San Salvador	Santa Ana	Managua	Chinandega	San Jose	Panama	Colon
N	464	300	428	294	471	289	733	518	317	3,814
Self-identification										
Heterosexual	4.53	5.33	7.24	22.79	2.34	6.23	13.23	4.25	2.21	7.60
Bisexual	51.08	46.00	34.58	30.61	24.63	41.18	50.20	23.36	15.14	36.31
Homosexual	38.36	43.67	45.79	30.27	49.04	36.33	34.38	65.06	69.09	45.57
Transvestite	6.03	5.00	12.38	16.33	23.99	16.26	2.18	7.34	13.56	10.50
Age category										
18–24	49.35	67.67	67.29	65.99	77.92	66.78	50.48	56.76	34.38	58.91
25–29	19.18	18.33	18.22	15.65	13.59	21.45	24.69	22.20	31.86	20.74
30–34	15.30	6.67	10.05	9.86	6.16	9.34	14.60	11.39	20.50	11.80
35 and higher	16.16	7.33	4.44	8.50	2.34	2.42	10.23	9.65	13.25	8.55
Marital status										
Single	79.74	87.67	86.68	89.12	83.23	79.24	84.45	77.41	71.61	82.17
Married	11.85	6.33	12.38	9.18	15.71	18.34	11.05	21.24	26.81	14.60
Separated/divorced/widowed	8.41	6.00	0.93	1.70	1.06	2.42	4.50	1.35	1.58	3.22
Educational level										
Primary or less	22.63	7.67	7.01	11.56	7.01	13.15	25.10	1.74	3.79	12.27
Secondary	30.39	15.00	24.30	24.49	71.13	76.12	57.71	70.85	64.67	50.13
Higher	46.98	77.33	68.69	63.95	21.87	10.73	17.19	27.41	31.55	37.60
Ethnicity										
Black	2.16	0.33	0.47	4.76	0.85	1.38	2.18	22.39	40.69	7.76
Indigenous	21.55	35.00	0.23	3.06	1.06	1.04	1.23	5.60	7.89	7.50
Ladino	74.78	64.00	92.99	87.76	96.18	91.35	28.51	46.53	41.64	65.39
White	1.51	0.67	6.31	4.42	1.91	6.23	68.08	25.48	9.78	19.35
Housing security										
Changed residences 2+ times in the past year	36.64	32.00	23.83	13.61	26.54	17.99	45.70	32.43	12.62	29.57
Socio-economic status										
Low	50.86	26.33	55.14	67.01	63.91	82.35	54.16	62.36	60.88	57.68
Middle	28.23	36.00	30.61	26.19	28.87	14.53	28.10	27.61	31.86	28.19
High	20.91	37.67	14.25	6.80	7.22	3.11	17.74	10.04	7.26	14.13

*Unweighted univariate values are calculated for an analytical sample with no missing values on all covariates.

Many of the HIV risk behaviors that we used as dependent variables in this analysis may be co-occurring. To account for this, a second set of models for each outcome was created in which the potentially co-occurring risk behaviors under study are included as independent variables (along with the original set of covariates). These are presented in column (II) under each outcome variable in Table 4. A similar analysis was used elsewhere (42) when examining co-occurring psychosocial health conditions as independent correlates of each other in multivariable models. This second set of statistical models allows us to determine whether there is a relationship between violence and each risk behavior, after accounting for the effects of other co-occurring risk behaviors. For the models associated with multiple partners and transactional sex, a high correlation and insufficient sample size in all cells required omission of the corresponding variable from the model as an independent variable (i.e. multiple partnerships were excluded as an independent variable in the transactional sex model and vice versa).

**Table 4:** Multivariate results of experience of violence on HIV risk behaviors, condom and lubricant use, and STI symptoms among MSM and transgender women in nine cities in Central America

	Transactional sex in the past 30 days		Multiple partners in the past 30 days		Sex under the influence of alcohol/drugs in the past 30 days		Condom+lubricant use at last sex		STI symptoms or diagnosis in the past 12 months	
				
	(I)	(II)	(I)	(II)	(I)	(II)	(I)	(II)	(I)	(II)
	(n=3,829) R^2^=0.1144 H-L p=0.0935	(n=3,829) R^2^=0.1379 H-L p=0.1504	(n=3,829) R^2^=0.075 H-L p=0.3392	(n=3,829) R^2^=0.1413 H-L p=0.7543	(n=3,829) R^2^=0.052 H-L p=0.099	(n=3,829) R^2^=0.120 H-L p=0.184	(n=3,784) R^2^=0.061 H-L p=0.269	(n=3,783) R^2^=0.065 H-L p=0.310	(n=3,829) R^2^=0.088 H-L p=0.301	(n=3,829) R^2^=0.094 H-L p=0.361
Psychological/verbal violence	1.112 [0.935, 1.345]	1.056 [0.878, 1.271]	1.120 [0.947, 1.325]	1.030 [0.863, 1.229]	1.359*** [1.164, 1.586]	1.349*** [1.147, 1.587]	1.017 [0.871, 1.187]	1.016 [0.869, 1.187]	1.797*** [1.448, 2.230]	1.742*** [1.403, 2.165]
Physical/sexual violence	1.761*** [1.421, 2.183]	1.643*** [1.320, 2.045]	1.368** [1.092, 1.713]	1.234 [0.974, 1.563]	1.509*** [1.242, 1.833]	1.370** [1.116, 1.682]	0.865 [0.710, 1.056]	0.838 [0.686, 1.024]	1.724*** [1.343, 2.213]	1.639*** [1.274, 2.110]
Self-identification (homosexual Ref.)										
Heterosexual	1.059 [0.762, 1.470]	0.953 [0.682, 1.332]	1.443* [1.065, 1.956]	1.285** [0.936, 1.764]	1.523** [1.154, 2.010]	1.414* [1.058, 1.890]	0.653** [0.492, 0.868]	0.649** [0.488, 0.864]	0.879 [0.580, 1.331]	0.843 [0.556, 1.279]
Bisexual	1.053 [0.861, 1.288]	0.977 [0.796, 1.200]	1.992*** [1.669, 2.377]	1.898*** [1.578, 2.282]	1.381** [1.171, 1.628]	1.176 [0.988, 1.400]	0.734*** [0.623, 0.865]	0.729*** [0.618, 0.861]	0.887 [0.700, 1.127]	0.846 [0.664, 1.078]
Transgender	3.940*** [3.066, 5.113]	3.769*** [2.891, 4.915]	1.789*** [1.372, 2.332]	1.667** [1.265, 2.198]	1.471** [1.159, 1.866]	1.141 [0.886, 1.468]	1.331* [1.051, 1.686]	1.222 [0.961, 1.555]	0.655* [0.461, 0.931]	0.613** [0.428, 0.877]
Transactional sex in the past 30 days				* excluded		1.648*** [1.389, 1.956]		1.447*** [1.217, 1.721]		1.099 [0.863, 1.400]
Multiple partners in the past 30 days		* excluded				3.799*** [3.157, 4.571]		1.039 [0.880, 1.227]		1.198 [0.930, 1.543]
Sex under the influence of alcohol/drugs in the past 30 days		2.313*** [1.959, 2.731]		4.419*** [3.704, 5.272]				0.888 [0.766, 1.029]		1.389** [1.128, 1.711]

All logistic regression models control for the following variables: age, marital status, having one or more children, socio-economic status (SES), educational attainment, ethnicity, city of residence, and housing insecurity.

Column (I) includes only violence, sexual identity, and the control variables listed above. Column (II) includes these and other relevant sexual risk behaviors.

* p<0.05

** p<0.01;

*** p<0.001.

## Results

### Univariate

The experience of violence varied greatly by type of violence and by city under study. MSM in Guatemala City reported the highest level of psychological violence (35.6%) and verbal violence (44.9%), while the cities of Panamá and Colon reported the lowest percentages of these types of violence (10.7 and 12.2% experienced psychological and 7.6 and 6.1% experienced verbal violence, respectively).

Guatemala City, Managua, and San Salvador presented the highest rates of physical violence (25.0, 18.3 and 16.0%, respectively). Regionally, 5% of men reported sexual violence – city-level estimates ranged from 7.4% in Managua to 2.0% in Chinandega.

There are also marked differences in the city-level estimates of reported risk behaviors. Regionally, 35.5% of MSM reported having sex under the influence of alcohol and/or drugs, with city-level variations ranging between 57.0% in Colon and 25.4% in Managua. Transactional sex was higher in the Capital cities (40.3% in Guatemala City and 35.8% in San Jose) as compared to the smaller cities (12.8% in Quetzaltenango and 17.3% in Colon). Regionally, 68.5% of MSM reported multiple partnerships in the past 12 months – more than half of MSM in all cities reported this risk behavior.

With regards to condom plus lubricant use, close to 50% of MSM regionally reported using protection at last sex – the lowest estimates are found in the two cities studied in El Salvador (San Salvador: 35.2%; Santa Ana: 37.9%) and Nicaragua (Managua: 39.0%; Chinandega: 38.3%). Reported STI symptoms or diagnosis were highest in Colon (34.6%) and lowest in the two cities studied in Nicaragua (Managua: 6.0% and Chinandega: 5.2%).

### Multivariate

The experience of physical violence (including sexual violence) within the past 12 months was significantly associated with all the HIV risk behaviors under study, after controlling for other factors. MSM who experienced physical violence were more likely to be engaged in transactional sex (OR: 1.76 [1.42–2.18]), have multiple partners in the past 30 days (OR: 1.37 [1.09–1.71]); and have engaged in sex under the influence of alcohol or drugs (OR: 1.51 [1.24–1.83]). The experience of psychological and/or verbal violence within the past 12 months was only found to be significantly associated with having engaged in sex under the influence of alcohol and drugs (OR: 1.36 [1.16–1.59]), but not with the other risk behaviors examined.

Another factor found to be associated with risk behaviors was the respondent's self-identification. Self-identification corresponds to the respondents reported sexual identity, which may also indirectly capture variables that are unmeasured by this study, including disclosure, internalized stigma, or experiences of social stigma. As compared to individuals who identified as being homosexual, TW were 3.9 times more likely to report engaging in transactional sex. Respondents who were heterosexual, bisexual, or transgender were also more likely to both report multiple partnerships (OR: 1.44 [1.07–1.96]; 1.99 [1.67–2.38]; 1.79 [1.37–2.33], respectively) and more likely to report engaging in sex under the influence of alcohol or drugs (OR: 1.52 [1.15–2.01]; 1.38 [1.17–1.63]; 1.47 [1.16–1.87], respectively) as compared to those identifying as homosexual.

In models controlling for other risk factors, the odds of reporting a risk behavior among those experiencing violence are attenuated (i.e. the odds ratios for violence in each model decrease when controlling for other risk factors), but in most cases the patterns of association remain significant. For example, after controlling for multiple partnerships and engagement in transactional sex, the odds of engaging in sex under the influence of alcohol/drugs for those experiencing physical violence are reduced from OR=1.51 [1.24, 1.83] to OR=1.37 [1.12, 1.68]. A similar pattern is seen with all other outcomes, with the exception of the model for multiple partnerships. In this case, the association between physical violence and multiple partnerships in the past 30 days goes from being significant to insignificant when controlling for having sex under the influence of alcohol/drugs. These results demonstrate that there is generally an independent association between violence and risk behaviors after controlling for other measured co-occurring risk behaviors.

Both physical violence and psychological/verbal violence were also associated with reporting STI symptoms or diagnosis within the past 12 months (OR: 1.72 [1.34–2.21] and 1.80 [1.45–2.23], respectively). In this case, self-identification as transgender had a protective effect (OR: 0.66 [0.46–0.93]) on reporting an STI. The association between violence and condom and water-based lubricant use was examined to determine if experience of violence had a negative influence on protective behaviors for HIV prevention. Here, no association was seen between either measure of violence and condom plus water-based lubricant use.

## Discussion

Guatemala and El Salvador present the highest reported levels of violence of all the cities included in this study. This finding is consistent with reports of violent crime among the general population across all countries in the isthmus (14). A major contribution of this study is the quantification of the experience of violence among MSM and TW in Central American cities, both as an important factor in HIV prevention and access to clinical services (43–46), and also from a human rights perspective. Estimates of sexual violence in MSM in Guatemala City are similar to comparable indicators among ever-married women (5.8% vs. 4.0%, respectively) (16). A similar pattern is observed in San Salvador: experience of sexual violence in the past 12 months in MSM is 6.5% compared to 4.5% among ever-married women (47). Our lack of data on perpetration makes comparing other indicators of violence between MSM and other populations difficult. However, our estimates of different forms of violence for MSM populations in Central America are similar to those reported for intimate partner battering by MSM in the United States (26). While this study and others have started documenting the high rates of violence experienced by MSM and TW in Central America (25, 48), the lack of official statistics and absence of political will that protects and recognizes key populations makes changing legislation, providing services, and documenting violence among these groups challenging.

There is a lack of homogeneity in the experience of violence and risk behaviors both across cities and according to sexual identification. As compared to those self-identifying as homosexual, respondents who report as transgender were more likely to have multiple partnerships and more likely to have had sex under the influence of alcohol and/or drugs. Our findings are in line with other studies that demonstrate that TW are more vulnerable, at greater risk, and have poorer health outcomes than MSM (49). In this study, and elsewhere, TW are several more times likely to engage in transaction sex than MSM (22).

We found that the experience of physical violence is associated with transactional sex and multiple partners and that both physical and psychological violence are associated with sex under the influence of alcohol or drugs. Though the associations were found to be strong in many cases (odds ranging from 1.5 to 1.7), the mechanism by which violence influences risk behaviors cannot be discerned and it is plausible that, just as violence can be a determinant of risk behavior, the risk behaviors measured in this study may also put individuals in a situation where they may experience violence. For example, this study examines whether the experience of violence influences engagement with multiple sexual partners, whereas other studies have examined engagement in multiple partnerships as a determinant of violence (50). In the experience of MSM in Central America, where individual risk behaviors take place within a context of homophobia, machismo culture, widespread gender-based violence, and widespread societal violence, it is likely that many risk behaviors and determinants of risk behaviors are co-occurring and reinforcing. In this study, we attempted to control for co-occurring risk factors to isolate the association between risk behaviors and violence. In doing so, we found the effects of violence attenuated, but not eliminated for most outcomes. The relationships between the co-occurring risk factors examined in this study have previously been described as a mutually enhancing syndemic (42), where epidemics are not only simultaneous but also interact and reinforce causing poorer health outcomes, which are sustained through social conditions. For example, substance abuse, violence, and HIV are described as phenomena that are concurrent and interdependent in how they influence health and well-being (18). For HIV prevention and care programs operating in this region, these results indicate that interventions cannot be implemented in isolation and must use combination prevention strategies that address violence prevention and care, as well as substance abuse, in order to truly address HIV risk behaviors. Our findings suggest that additional social and behavioral studies that deepen our understanding of the interplay between violence, alcohol abuse, drug use and mental health disorders are necessary to better address HIV risk behaviors in this population. Individual-level interventions may be effective at reducing risk behaviors of MSM and TW; however, these effects are stifled by the absence of higher order structural changes, within social networks, communities, and policies (23). Political will and legislation that is inclusive of MSM and TW is imperative to give visibility and ensure their full inclusion in the HIV prevention response (4). Future interventions should work toward ensuring legislation of protective laws and access to services such as physical and mental health care, protection, security, and legal representation.

A key outcome under this study was having sex under the influence of alcohol and/or drugs within the past 30 days. This outcome is relevant under the premise that abuse of alcohol and drugs influence the overall use and, more specifically, correct use of condoms during high-risk encounters. The co-occurrence of alcohol abuse and high-risk sex signals other potential conditions and behaviors of poor health such as violence and mental health disorders. Alcohol abuse is also an important factor because it is not only associated with victimization but also with perpetration of violence (51). In many contexts, studies among women show associations between alcohol abuse and experiencing intimate partner violence (52). This same pattern is repeated among studies examining interpersonal violence by MSM in the United States (7). Previous studies that examine the associations between violence, risk behaviors, and HIV have found that depression and violence have a strong association with HIV status that is mediated by risk behaviors, including drug and alcohol abuse (50, 53, 54). Similar results are found in research conducted among young, urban MSM (55), where psychosocial health problems (such as binge drinking, drug use, and interpersonal violence) were found to have additive effects on the odds of HIV positive status and on more proximal sexual risk behaviors, including multiple anal sex partners and unprotected anal sex.

Absent from our findings is a significant association between violence and condom and water-based lubricant use at last sex. The theoretical model framing this study postulates that the experience of violence interferes with condom use and access to health care and health products (46). We hypothesize an inverse association between the experience of violence and condom plus water-based lubricant use; however, no such association is observed. It is likely that the absence of information regarding the identity of perpetrators of violence (whether an intimate partner or some other person) and whether the perpetrator corresponds to the current sexual partner may inhibit this study from capturing the reduced agency of victims of violence within the context of an abusive relationship.

## Limitations

Over the past decade, RDS has become a methodology commonly used to draw samples of hidden populations for which traditional sampling frames do not exist (56). Recently, studies have indicated that RDS estimates have high variances and likely do not meet all the assumptions underlying the methodology (57, 58). Critics of the methodology rightly call for caution when implementing and interpreting RDS studies as, in some cases, they may be inadequate to meet some of the objectives for which they are used. While caution must be used in interpreting results, RDS is one of the limited methods available to collect data on MSM in Central America, and presents advantages over alternatives with regards to reaching important populations subgroups, ensuring containment of costs and other practical considerations, such as ensuring safety of teams during field work (59). We attempted to reduce these sources of bias by ensuring that initial seeds were diverse with regards to variables deemed important to recruitment. During analysis, we also verified that all samples achieved the maximum number of waves to reach equilibrium and that homophily within the sample was within a tolerable range.

Pooled data across all cities were used to conduct multivariate analysis. Previous studies conducted with RDS have pooled data across samples for this purpose, some of which have attempted to control for the clustered nature of the data and dependence of observations (39, 60–67). The unavailability of MSM population-size estimates for each city under study and the independence of our RDS samples (which are not linked across cities) precluded us from implementing this strategy. By presenting unweighted estimates from the pooled data, we acknowledge that the results, though presented as regional, do not represent regional estimates of MSM in Central America.

Another important limitation of this study is the cross-sectional nature of the data, which does not allow us to establish a temporal order between outcomes and exposure to violence. Even though the recall period for variables measuring violence is 12 months, and the recall period for most associated behaviors is 30 days, we still cannot assume that the reported experience of violence preceded that of the reported risk behaviors. The study used a 30-day recall period for measures of self-reported condom use, substance abuse, and transactional sex to minimize recall bias.

Results of this study may be subject to omitted variable bias in the models predicting risk behaviors. There is the potential that key variables that may be associated with the outcomes under study, but also associated with the experience of violence, such as depression or childhood experience of violence, are unmeasured and therefore unaccounted for in our results. Finally, this study lacks information regarding the relationship between respondents and perpetrators. Absence of this information has implications in the interpretation of findings and the operationalization of results in the HIV prevention context as acts of violence perpetrated by intimate partners may require intervention that differs from acts of violence perpetrated by others.

## Conclusion

Victimization of MSM and TW by different forms of violence is widespread in Central American cities. The experience of violence is shown in this study and in previous research to be independently associated with risk behaviors for HIV infection. Though the association between violence and other factors of risk and vulnerability, such as drug and alcohol abuse, are not fully understood, their catalytic effects on HIV risk behaviors require consideration in HIV prevention and human rights programs that work with these populations. The high prevalence of violence experienced by MSM and TW coupled with a lack of formal structures in Central American countries to report and follow-up cases of violence, forces survivors to remain silent. Our findings provide evidence that can be used to raise awareness and make policy level, structural, and programmatic changes to address these challenges.
